# Oxidative Stress as a Possible Target in the Treatment of Toxoplasmosis: Perspectives and Ambiguities

**DOI:** 10.3390/ijms22115705

**Published:** 2021-05-27

**Authors:** Karolina Szewczyk-Golec, Marta Pawłowska, Roland Wesołowski, Marcin Wróblewski, Celestyna Mila-Kierzenkowska

**Affiliations:** Department of Medical Biology and Biochemistry, Ludwik Rydygier Collegium Medicum in Bydgoszcz, Nicolaus Copernicus University in Toruń, 24 Karłowicza St, 85-092 Bydgoszcz, Poland; karosz@cm.umk.pl (K.S.-G.); marta.pawlowska@cm.umk.pl (M.P.); roland@cm.umk.pl (R.W.); marcin.wroblewski@cm.umk.pl (M.W.)

**Keywords:** antioxidants, drugs, oxidative stress, pathogenic process, *Toxoplasma gondii*, toxoplasmosis treatment

## Abstract

*Toxoplasma gondii* is an apicomplexan parasite causing toxoplasmosis, a common disease, which is most typically asymptomatic. However, toxoplasmosis can be severe and even fatal in immunocompromised patients and fetuses. Available treatment options are limited, so there is a strong impetus to develop novel therapeutics. This review focuses on the role of oxidative stress in the pathophysiology and treatment of *T. gondii* infection. Chemical compounds that modify redox status can reduce the parasite viability and thus be potential anti-*Toxoplasma* drugs. On the other hand, oxidative stress caused by the activation of the inflammatory response may have some deleterious consequences in host cells. In this respect, the potential use of natural antioxidants is worth considering, including melatonin and some vitamins, as possible novel anti-*Toxoplasma* therapeutics. Results of in vitro and animal studies are promising. However, supplementation with some antioxidants was found to promote the increase in parasitemia, and the disease was then characterized by a milder course. Undoubtedly, research in this area may have a significant impact on the future prospects of toxoplasmosis therapy.

## 1. Introduction

*Toxoplasma gondii* is a worldwide distributed obligate intracellular protozoan pathogen of humans and more than 30 species of birds and 300 species of mammals [1]. It remains the best model system to study the phylum *Apicomplexa*, which includes a number of unicellular parasites that are causative agents of several diseases, such as malaria, babesiosis and cryptosporidiosis [2]. All the apicomplexan parasites invade the cells; thus, they have to adapt to the host cell environment and evade the host response. An important element to combat all invading pathogens is the oxidative burst induced by the immune system, so the apicomplexans have to deal with oxidative stress inside their host cells [3].

*T. gondii* causes toxoplasmosis, which is one of the most common parasitic infections in humans. The prevalence of the disease ranges between 30%–50% of the world’s human population [1]. Toxoplasmosis is a well-known disease entity, and the biology of the parasite is described quite well. Nonetheless, the disease continues to occur with high frequency in the human population and still presents a risk of severe (fatal) disease in some patients. Despite significant progress made in the field of the therapy of human diseases, few advances have been made in the treatment of toxoplasmosis [4]. Available treatment options for toxoplasmosis patients are limited, which underscores the urgent need for searching for new, more effective therapeutics for both the acute and latent forms of the infection. The development of host-targeted adjunctive therapies that could provide new options for toxoplasmosis treatment and reduce the risk of drug resistance seems to be of first importance. The contribution of pro- and antioxidant processes to toxoplasmosis, and its treatment was previously described [5,6,7]. Thus, the use of oxidant and/or antioxidant compounds against the parasite should be strongly considered. So far, the therapeutic effects and safety of drugs against *T. gondii* infections, including oxidative effects, have been investigated mainly on animal models, most commonly in mice [8]. The available data are still sparse.

Taking into account the relevance of toxoplasmosis as a healthcare issue, this article aims at reviewing the current state of knowledge on the involvement of oxidative stress in the course of the disease and in the host defense, as well as the antioxidant system in the parasite organism. Additionally, the presented review provides new scientific data on the antioxidant mechanisms of action of drugs used in the treatment of toxoplasmosis. Potent candidates for toxoplasmosis treatment, whose mechanisms of action are attributed to the oxidant–antioxidant balance both in the parasite organism and in its host, as well as the potentially supportive action of antioxidant supplements, are discussed. We would like to inspire scientists to further research on the use of compounds modifying oxidative stress in the treatment of toxoplasmosis.

## 2. Characteristics of *Toxoplasma gondii*

Toxoplasma gondii was first described in 1908 by Nicolle and Manceaux in the gundi, a small hamster-rodent of Ctenodactylidae [9]. The researchers named it Toxoplasma gondii basing on its morphology (modern Latin: toxo—arc or bow, plasma—life) and the host. In retrospect, the correct name for the parasite should have been T. gundi, but Nicolle and Manceaux identified the host as Ctenodactylus gondi instead of Ctenodactylus gundi, which is a correct name [9]. There are three main genotypes of Toxoplasma gondii (type I, II and III), with different virulence and epidemiological patterns, which largely predominate in Europe and North America [10,11]. Apart from these three main clonal lineages, there are also atypical genotypes, more complex and with higher genetic diversity. Dardé [11] suggests that there are relationships between the genotype and human disease, but these mechanisms are still poorly understood. Recombination may lead to acquiring new pathogenic mechanisms of Toxoplasma and manifest in humans by a severe course of toxoplasmosis, also in immunocompetent patients.

### 2.1. Life Cycle

The Toxoplasma gondii life cycle includes sporogony (sexual reproduction in definitive hosts) and schizogony (asexual reproduction in intermediate hosts). Cats are the only definitive hosts of this parasite and become infected by eating meat (mostly rodents, birds, or slaughterhouse remains) containing tissue cysts or by ingesting oocysts from the soil [12]. Bradyzoites released from tissue cysts form schizonts in the intestine of cats. After merulation, schizonts release merozoites. Only in cats can the parasite form gametocytes, which further develop into gametes [13]. After fertilization, they form oocysts, which are shed in feces. However, only sporulated oocysts containing sporozoites become infective for intermediate hosts [14]. In intermediate hosts, upon oral uptake, sporozoites transform into the invasive tachyzoite stage. Tachyzoites are proliferative, fast-multiplying forms of the parasite. They can multiply in a variety of cells and eventually encyst in several tissues, particularly in the brain [15]. Tissue cysts can be found in the retina, brain, skeletal and heart muscle [16]. They are the infective stages for intermediate and definitive hosts [15]. Humans can be infected by eating meat containing tissue cysts, which are resistant to gastric digestion and thus infectious orally, whereas tachyzoites are destroyed by gastric juice [12]. Infective tachyzoites develop from bradyzoites, which are released from lysed cysts in the intestine. Rapidly multiplying tachyzoites can transform to metabolically inactive bradyzoites and form tissue cysts. Tissue cysts may remain dormant for a long time, perhaps for the life of the host [13]. Occasionally, rupturing tissue cysts release bradyzoites, which are killed in immunocompetent hosts. However, in immunodeficient hosts, bradyzoites released from tissue cysts may multiply locally and spread to other organs [14,15].

### 2.2. Routes of Toxoplasma Infection in Humans

To summarize, humans can be infected by three different forms of the parasite, namely tachyzoites, bradyzoites and sporozoites. Oocysts of Toxoplasma, formed in the intestine of Felidae (cats), are excreted in the cat’s feces into the environment. About 2 or 3 days after excretion, oocysts undergo sporulation and become an infective form containing sporozoites [13,14,17]. These forms are resistant to environmental factors and can stay infective for more than one year [13]. It should be noted that humans can be infected by ingestion of vegetables, fruit or water contaminated with sporozoites, but contrary to appearances, it is not the most common way of getting infected. More commonly, people become infected by eating raw, cured, dried, smoked or undercooked meat, particularly lamb and pork, containing tissue cysts with bradyzoites [13,14,17,18,19]. Chicken meat is a source of infection for cats, but also for humans if eaten undercooked [18]. It should be considered that chickens are one of the most important hosts of toxoplasma. The prevalence of toxoplasmosis in chickens raised in backyards reaches up to 100% because they feed on potentially contaminated ground [18]. This makes pasture chickens a significant risk factor for Toxoplasma infection. However, studies on experimentally infected hens suggest that chicken eggs should not be considered as a source of infection [18]. In a case-control study, Jones et al. [19] identified eating raw mollusks, namely oysters, clams and mussels, as a new risk factor for Toxoplasma infection. As mollusks are filter feeders, the parasite concentrates in their organisms. Another potential source of transmission is un-pasteurized milk and milk products [14,17]. Toxoplasma gondii has multiple pathways to infect a human organism, apart from those mentioned. Tachyzoites can be transmitted also by blood transfusion. Congenital toxoplasmosis, which is an effect of vertical transmission of tachyzoites from mother to fetus through the placenta, should be also mentioned [13,14].

### 2.3. Prophylaxis of Toxoplasma Infection in Humans

In relation to the main sources of the parasite and routes of Toxoplasma transmission, the prevalence of the disease reflects mainly hygienic and dietary practices of human populations [13]. To protect the soil against contamination with sporozoites, litter boxes should be used by cat owners. As it takes several days for oocysts to sporulate, litter boxes should be cleaned up daily, but not by people from increased risk groups, such as pregnant women, or immunosuppressed people [19]. Although there are effective and simple methods to inactivate cysts of the parasite in meat, for example, deep-freezing (−12 °C or lower for at least 24 h), low social awareness of the disease causes Toxoplasma eradication still ineffective. Most people are aware of the association of toxoplasmosis with cats, but they are rather unaware of exposure to the parasite during their daily activities, especially the risk associated with undercooked meat [19]. It should be borne in mind that the parasite can also be transmitted if care is not taken to wash hands thoroughly after cutting meat and during meat cooking [18]. Hence the need arises to educate society about the risk of toxoplasmosis transmission, among others, by popularizing safe-cooking guidelines and to take into account other sources of the parasite in toxoplasmosis prevention. Reduction of T. gondii contamination of meat and increased education of health professionals and the public regarding risk factors could help to further reduce the burden of toxoplasmosis [19]. 

### 2.4. Toxoplasmosis Course in Humans

There are three clinical forms of the disease, including ocular, congenital and cerebral ones. Toxoplasma gondii is potentially responsible for significant morbidity and mortality in the congenitally infected fetus (especially in the first trimester of pregnancy) because it can cause a serious disease course and even miscarriage. In a congenital disease course, hepatitis and pneumonia may be followed by central nervous system involvement resulting in hydrocephalus, retinochoroiditis and cerebral calcifications [10]. In immunocompetent humans, the disease is said to be latent (clinically asymptomatic), but the disease course can be severe in immunocompromised or immunosuppressed patients [10,13]. In an oligosymptomatic course, the disease may produce mild, flu-like illness. In less than 10% of cases, it causes mononucleosis-like syndrome with headache, malaise, fever, cervical lymphadenopathy and fatigue [13]. Some tachyzoites can evade the immune response or drugs used in the treatment and transform into bradyzoites inside quiescent cysts, long-term forms of the parasite. Additionally, Toxoplasma can use immune cells to migrate and infiltrate the brain. Recent investigations indicate that toxoplasmosis may increase the risk of brain cancer [13,20]. In the brain, Toxoplasma prefers neurons because these cells do not react to inflammatory cytokines. Thus, a strong anti-parasitic immune response is not induced [13]. As a result, central nervous system (CNS) toxoplasmosis, or toxoplasmic encephalitis (TE), which is the most severe (even fatal) course of the disease, may develop in immunocompromised individuals. Encephalitis is the predominant clinical manifestation of toxoplasmosis in acquired immune deficiency syndrome (AIDS) patients and is believed to be due to the reactivation of latent infections [15]. Clinically, the most common manifestations of TE are neurological abnormalities and brain abscesses accompanied by fever [13]. Interestingly, Toxoplasma can change the behavior of hosts, including humans. Infected rodents experience less fear of feline odors, which makes them more likely to be eaten by the parasite definite host [21]. The possible relationship of Toxoplasma infection with mental illness in humans has focused the attention of many researchers [22]. There have been numerous studies on T. gondii infection and its impact on human behavior and mental diseases [23]. Nonetheless, the effect of the parasite on human behavior is still less certain than in rodents [24]. The influence of T. gondii on the increased probability of developing psychiatric disorders, including schizophrenia, bipolar disorder and obsessive-compulsive disorder, has been confirmed [21]. However, no parasite genes or effector proteins responsible for parasitic behavioral changes have been identified [21]. Postulated mechanisms linking toxoplasmosis and behavior include generalized inflammation, alteration in endocrine signaling and changes in neurotransmitter pathways [24]. The main weakness of the studies conducted so far is that they rely on the correlation between T. gondii seropositivity and behavioral or cognitive outcomes but do not assess directly the causal role of the infection in a given pattern of behavior [23]. Taking into account study limitations and the results of meta-analyses, it could be suggested that the effect of the parasite on human behavior or disease might be rather mild. It is likely that the influence of T. gondii on human behavior and mental health may be dependent on a number of factors, including the parasite strain type, route and duration of infection, host genotype, and others [23]. Undoubtedly, more research is needed in this area.

## 3. Oxidative Stress in *Toxoplasma gondii* Infection

Oxidative stress is defined as an imbalance between the production of reactive oxygen species (ROS) and the antioxidant system of the organism [25,26]. ROS include all highly reactive and unstable derivatives of molecular oxygen, such as hydrogen peroxide (H_2_O_2_), superoxide anion (O_2_^−^) and the most dangerous hydroxyl radical (OH^∙^), which is formed in the Fenton and/or Haber–Weiss reaction [27,28]. ROS homeostasis is closely related to many other reactive molecules, such as reactive carbonyl species (RCS) and reactive nitrogen species (RNS) [29]. Since ROS are produced as a consequence of oxygen metabolism, it is impossible to avoid them in aerobic organisms. They are generated in the cytosol and in such organelles, such as mitochondria and peroxisomes [30,31]. At physiological levels, ROS are involved in cell signaling processes, but enhanced oxidative stress due to the excessive ROS formation may cause damage to all cellular macromolecules such as lipids, proteins and nucleic acids, ultimately leading to cell death [7,31,32]. The characteristic action of ROS is the degradation of polyunsaturated fatty acids in the process termed lipid peroxidation, which leads to the production of harmful molecules, including malondialdehyde (MDA) [33]. MDA is a highly reactive aldehyde that causes oxidative damage in tissues. MDA belongs to thiobarbituric acid reactive substances (TBARS) and is a major TBARS in the organism. Therefore, for the sake of method simplicity, the TBARS measurement is commonly used to assess the MDA concentration [34]. Both TBARS and MDA are considered to be the most important markers of oxidative stress and lipid peroxidation in biological samples [35]. 

### 3.1. Oxidant–Antioxidant Balance in Humans

ROS are related to a wide variety of human disorders, such as chronic inflammation, age-related diseases and cancer [36,37]. Apart from that, ROS are also essential for various biological functions, including cell survival, cell growth, proliferation and differentiation, and immune response [38,39]. In phagocytes, ROS are generally produced in a specific process with nicotinamide adenine dinucleotide phosphate (NADPH) oxidase or by the mitochondrial respiratory chain [31,40]. NADPH oxidase plays a vital role in inflammatory processes by catalyzing the production of the superoxide anion radical and excessive production of other ROS, leading finally to cellular damage. The resulting cellular damage alters the immune response to pathogens and ultimately modifies susceptibility to bacterial, viral and parasitic infections [35]. 

All oxygen-metabolizing cells are equipped with cellular antioxidants to counteract the harmful effects of ROS action. Detoxification and removal of deleterious oxidants are essential in biological systems to restore redox homeostasis of the cell [30,41]. The complex endogenous antioxidant system includes antioxidant enzymes such as superoxide dismutases (SODs), catalase (CAT), glutathione peroxidases (GPxs) and glutathione reductase (GR) [42,43], as well as non-enzymatic compounds such as reduced glutathione (GSH) and uric acid [44,45]. GSH protects the cell against the detrimental effects of endogenous and exogenous oxidants by conjugation of reactive species and detoxification of lipid peroxidation products [46,47]. Moreover, GSH prevents the conversion of hemoglobin into methemoglobin in the oxidation reaction [7,48]. At present, antioxidant vitamins A, C and E are considered to be the main exogenous antioxidants playing a significant role in neutralizing free radicals and maintaining oxidative homeostasis [49,50].

### 3.2. Role of Oxidative Stress in Toxoplasma Infection

**Figure 1:**
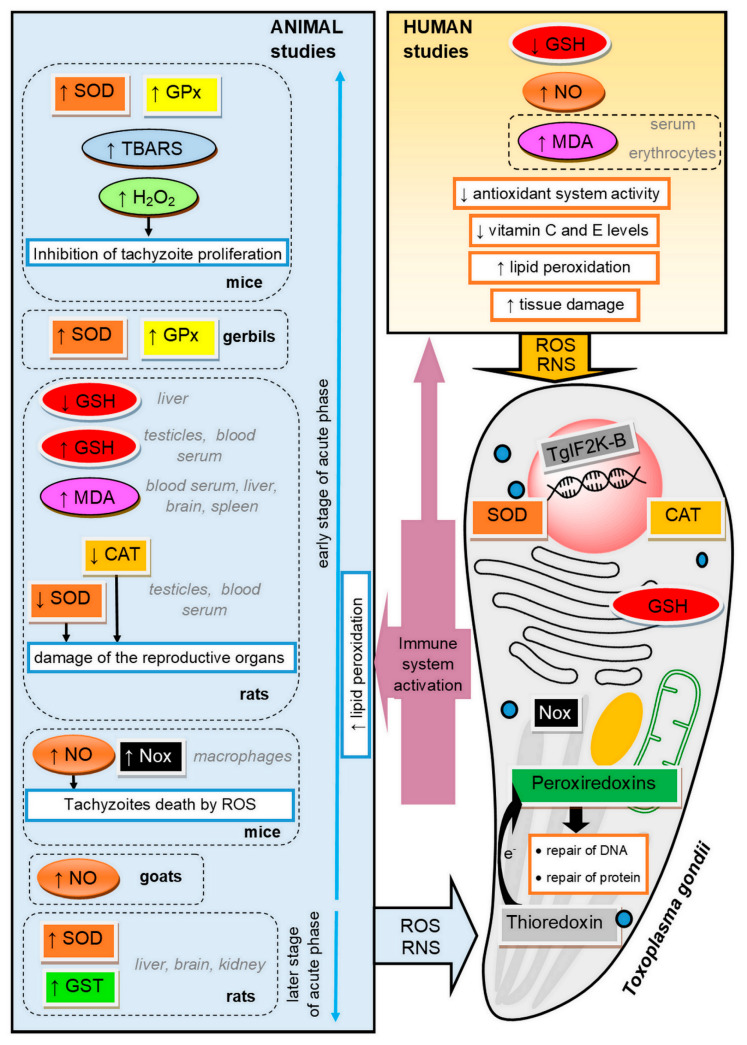
The mechanisms of oxidative injury and antioxidant defense in the course of toxoplasmosis in the parasite and in the host. SOD, superoxide dismutase; GPx, glutathione peroxidase; TBARS, thiobarbituric acid reactive substances; GSH, reduced glutathione; MDA, malondialdehyde; CAT, catalase; NO, nitric oxide; Nox, NADPH–oxidase complex; GST, glutathione S-transferase; RNS, reactive nitrogen species; ROS, reactive oxygen species; TgIF2K-B, *Toxoplasma gondii* eukaryotic initiation factor 2 (eIF2α) kinase.

Oxidative stress plays a significant role in the course of Toxoplasma infection in the organism of both the host and the parasite [51] (see Figure 1). When T. gondii multiplies asexually, it causes cellular disruption and cell death in an infected host. The resulting necrosis attracts inflammatory host cells, such as lymphocytes and monocytes. In the immune response against the parasite, enormous amounts of ROS and RNS are generated [41]. Oxidative stress resulting from the host response is toxic to parasites, but on the other hand, many studies reported that deleterious consequences of parasitic infection in a host organism are the result of defense mechanisms involving increased production of ROS [52]. The processes related to oxidative stress were proved to play a pivotal role in the pathogenesis of toxoplasmosis in animals and humans [53,54].

### 3.3. Antioxidant Defense of Toxoplasma gondii

ROS, generated in immune response, are able to kill various intracellular pathogens, including T. gondii. It was found that ROS can inhibit the activity of T. gondii in monocytes of infected animals. For example, high H_2_O_2_ concentration may inhibit the intracellular proliferation of tachyzoites [55]. This is why the parasite must protect itself against the oxidative burst imposed by the host [5,55,56]. Toxoplasma cells express SODs (one cytosolic and two mitochondrial), CAT and three peroxiredoxins, including one 1-Cys peroxiredoxin and two 2-Cys peroxiredoxins [5,55,57,58]. SODs and CAT belong to the primary antioxidants involved in the defense against oxidative stress and oxidative metabolic by-products [59]. SODs are metalloproteins that catalyze the dismutation of superoxide anions to form molecular oxygen and hydrogen peroxide [60]. CAT catalyzes hydrogen peroxide conversion to water and oxygen and thereby diminishes its cellular level [56,61]. The intrinsic kinetic properties of CAT and its cytosolic localization suggest that this enzyme might be best suited to the protection against host cell oxidative stress [5]. According to knockout studies, CAT seems to have an important role in invasion and replication inside the parasitophorous vacuoles [6]. T. gondii deficient in CAT exhibited increased susceptibility to exogenous hydrogen peroxide and was less virulent in mice [5]. Peroxiredoxins have phospholipase activity and are able to detoxify H_2_O_2_ in the presence of dithiothreitol [57]. Increased peroxiredoxin expression was found to be associated with the protection of the parasite against oxidative stress [6]. In organisms possessing CAT, the peroxiredoxins are unlikely to play a pivotal role in antioxidant defense, and recent studies suggest that these enzymes might fulfil quite distinct functions [5]. However, it seems likely that peroxiredoxins are important to T. gondii in view of the fact that the parasite is able to survive the knockout of catalase [57]. 

The classical antioxidant systems such as thioredoxins (Trx) and GSH are also suggested to occur in Toxoplasma, which is corroborated by the presence of trx genes in the transcriptome [62]. The role of the Trx system is to deliver electrons to the peroxiredoxins, which enables the removal of ROS and RNS. In addition, this system is involved in the repair of oxidative damage of DNA and proteins [63]. Thioredoxin reductase, which catalyzes the reduction of the oxidized form of thioredoxin with the consumption of NADPH, has the ability to maintain the Trx redox state, thus protecting the parasite against free radical damage in host immune cells [55].

### 3.4. Adaptative Response of Toxoplasma gondii to Oxidative Stress

Regardless of the variety of antioxidant systems present in T. gondii, tachyzoites are highly susceptible to exogenous and endogenous oxidative stress. Minor changes in the redox balance of the parasite cell can lead to the destruction of its oxidative homeostasis and ultimately its death [57]. In eukaryotes, environmental stress induces the integrated stress response via phosphorylation of translation initiation factors, including eukaryotic initiation factor 2 (eIF2) [64]. Interestingly, a similar adaptive mechanism was observed in T. gondii. Augusto et al. [65] demonstrated that the Toxoplasma eIF2 kinase TgIF2K-B is activated in response to oxidative stress and affords protection. The presence of TgIF2K-B enables the parasite to regulate its replication rate under oxidative stress as the activation of this enzyme influences the maintenance of oxidant–antioxidant balance in the parasite organism. For Toxoplasma, the critical moment of adaptation is the differentiation into bradyzoite cysts. Parasites lacking TgIF2K-B would likely have reduced transmission through the predation route due to their compromised ability to form infectious tissue cysts [65].

### 3.5. Antioxidant Defense of the Host during Toxoplasma Infection

The enzymatic antioxidant defense is one of the mechanisms that protect the host cells against an excess of free radicals due to parasitic infections [42,66]. Nazarlu et al. [67] observed a decrease in SOD and CAT activity in the blood serum, but also in the testes of rats infected with T. gondii. It was also found that the activity of SOD was reduced in erythrocytes of gerbils with toxoplasmosis [30,41], while Bahrami et al. [68] observed a significant increase in GPx activity in the blood of rats infected with T. gondii on the seventh day after the infection. 

Another compound that plays a relevant role in antioxidant mechanisms is GSH, as the thiol group of GSH transmits a reducing equivalent of ROS [69]. A decrease in GSH concentration may indicate the activation of antioxidant mechanisms in response to the T. gondii infection. Significant depletion in the GSH level, observed in rats infected with T. gondii, is supposed to be an important factor in toxicity, leading to host brain tissue damage and a reduction in the activities of antioxidant enzymes [68,70,71]. Similarly, Nazarlu et al. [67] observed decreased GSH levels in the blood serum and in the testes of rats infected with T. gondii. The authors believe that decreased GSH levels in chronic toxoplasmosis could change the detoxifying capacity of reproductive tissue and thus cause oxidative damage to reproductive organs and, consequently, affect adversely fertility. A decrease in GSH concentration was also detected in patients with toxoplasmosis, which suggests the occurrence of changes in the oxidant–antioxidant balance as a mechanism of tissue damage in cases of toxoplasmosis in humans [50,54]. The GSH action is suggested to be increased by vitamins C and E [54]. In addition to the enzymes, antioxidant vitamins are also involved in maintaining cell homeostasis and neutralizing free radicals. Vitamin C is a fundamental supplement involved in the repair of tissues, while vitamin E is a fat-soluble antioxidant interrupting the propagation of ROS [31,72]. Khaleel et al. [50] observed decreased levels of antioxidant vitamins in women with toxoplasmosis.

### 3.6. Oxidative Stress in the Early Stages of the Acute Phase of Toxoplasmosis

It is believed that, during the acute phase of toxoplasmosis, ROS are intensively produced, and oxidative stress is induced in the tissues of infected animals as the host defense against the infection. T. gondii seropositive cats, even when asymptomatic, show an increase in ROS levels [73]. The host immune response to infections typically involves phagocytosis after the activation of mononuclear phagocytes by lymphokines [74,75]. Although the NADPH–oxidase complex (Nox), which produces ROS, is expressed in almost every mammalian tissue, its function of producing ROS in host defense against pathogens is more pronounced in phagocytes [76]. Hence, the activation of parasiticidal mechanisms via ROS generation occurs mainly in host phagocytes. Nox-mediated ROS generation, as a factor involved in regulating the intracellular survival of T. gondii parasites within macrophages, was demonstrated in mice by Matta et al. [77]. 

An antiparasitic effect on protozoa was also revealed for nitric oxide (NO) derived from macrophages [78]. T. gondii infection is associated with an increased NO level, which is generated in an attempt to control the infection. Tonin et al. [79] noted an increase in NO levels in goats with toxoplasmosis. NO can directly cause the death of tachyzoites or stimulate the production of the heat-shock protein 70 in tachyzoites of both virulent and non-virulent strains, contributing to their conversion to the bradyzoite stage and cyst formation [79,80].

### 3.7. Oxidative Stress in the Later Stages of the Acute Phase of Toxoplasmosis

The vast majority of research has focused on describing oxidative stress during the early stages of the acute phase of toxoplasmosis. However, there are a few studies investigating the oxidant–antioxidant balance in the later stages of the acute phase of T. gondii infection. Türkoğlu et al. [81] observed increased SOD and glutathione S-transferase (GST) activities 30 days after infection in the brain, liver and kidney of rats. The researchers suggest that the increased antioxidant defense persisting later on in the acute phase can be used to diagnose T. gondii infection and may be helpful, especially in cases where the diagnosis of T. gondii infection is difficult in the earlier stage of the acute phase.

### 3.8. Oxidative Damage in the Host during Toxoplasma Infection

The rapid release of ROS and NO plays an important role against T. gondii, but also contributes to oxidative injury, inflicting tissue damage and the disease pathology [68,82,83]. Oxidative stress leads to intracellular lysosomal membrane damage, which is followed by apoptosis or necrosis [84]. The reduced activity of the defense system protecting tissue against free radical damage in Toxoplasma seropositive patients and animals is associated with increased lipid peroxidation [48,53]. Atmaca et al. [41] suggested that T. gondii induces lipid peroxidation in infected gerbils. In rats infected with T. gondii, an increased MDA concentration was observed in the liver cells [71], as well as in the blood serum and in the testes [67]. Moreover, [70] an increase in TBARS concentration was found in the brain of mice infected with T. gondii. Some authors reported a significant increase in the concentration of MDA in the serum of humans with chronic toxoplasmosis. Kiran et al. [47], Al-Kuraishy et al. [85] and Yazar et al. [53] reported that MDA levels were significantly higher in asymptomatic Toxoplasma seropositive patients compared to healthy subjects. There was no correlation between age or gender and the MDA concentration. An increased concentration of MDA was also observed in the erythrocytes of women with toxoplasmosis in comparison with the control group [48].

Despite the available literature data, more research on T. gondii infection in combination with other diseases is still needed to better understand the changes caused by this parasite in its host and to establish the diagnosis and appropriate treatment. 

## 4. Oxidant–Antioxidant Effects in Toxoplasmosis Treatment

The gold standard in toxoplasmosis treatment is the combination of pyrimethamine and sulfadiazine (pyr–sulf), targeting the active stage of infection [4]. There are also other therapies available, including pyrimethamine in combination with some antibiotics, as well as monotherapy with sulfamethoxazole-trimethoprim (ST) or atovaquone [8,86]. Nevertheless, none of these therapies was found to be superior to pyr–sulf, and no therapeutic is able to eliminate T. gondii cysts from the infected host [87]. The main targets of typical anti-Toxoplasma drugs are enzymes involved in the folate synthesis pathway, namely dihydrofolate reductase (DHFR) and dihydropteroate synthetase (DHPS) [88]. DHFR is also present in humans, so this therapy may result in a folic acid deficiency, which in turn is possibly responsible for severe hematological side-effects [86]. To date, all current therapeutical strategies have several limitations, including adverse severe side-effects and treatment failure because of drug resistance [89]. In this respect, there is an urgent need for searching alternative compounds with novel mechanisms of action. The numerous drug targets identified against Toxoplasma gondii include the inhibition of the mitochondrial electron transport chain, synthesis of fatty acids, isoprenoid pathway, synthesis of DNA, synthesis of proteins, as well as the action of Toxoplasma gondii calcium-dependent protein kinase 1 (TgCDPK1) [90,91]. In addition, targeted host-directed immunotherapy aimed at activating or suppressing specific elements of the immune system has been considered [91].

**Figure 2:**
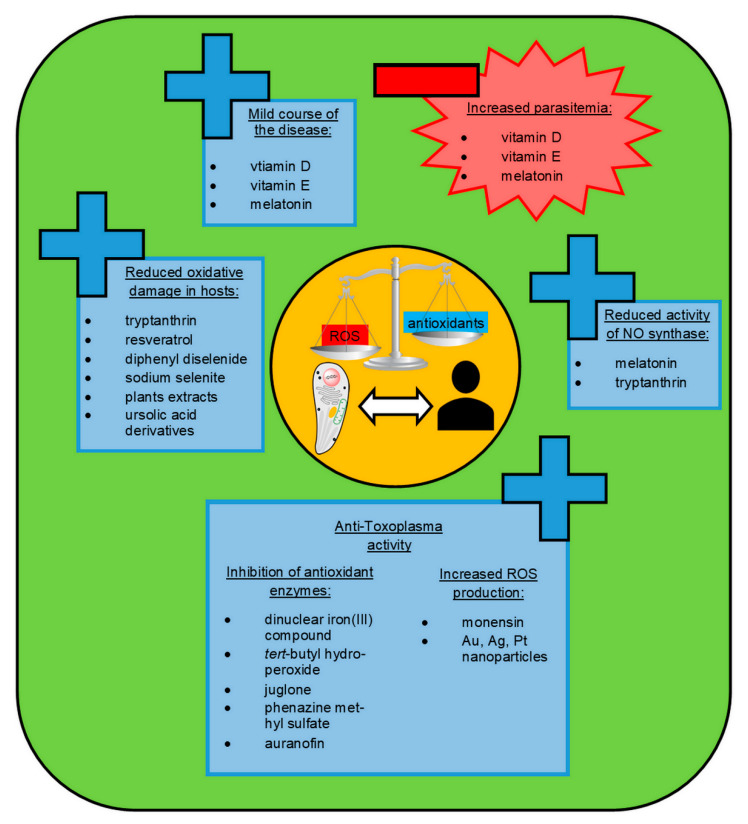
The chemical compounds that may affect the oxidant–antioxidant balance as promising novel anti-*Toxoplasma* therapeutics. ROS, reactive oxygen species; NO, nitric oxide.

In view of the research on the contribution of oxidative stress to the pathology of toxoplasmosis, the chemical compounds that affect the oxidant–antioxidant balance are of high interest as possible novel anti-*Toxoplasma* therapeutics (see Figure 2). The antioxidant system of *T. gondii* is known to play an important role as a defense mechanism against oxidative stress imposed by the host and to contribute as a virulence factor in vivo [5]. 

### 4.1. Inhibition of Antioxidant Defense of Toxoplasma gondii

The inhibition of antioxidant enzymes of the Toxoplasma parasite seems to be a viable target for the development of novel drugs against this pathogen. One of the antioxidant enzymes is CAT, which lacks in most pathogenic protozoans, but it is present in the T. gondii cytoplasm, where it neutralizes the peroxides produced by the host [6]. CAT and SOD seem to be the most important antioxidants of T. gondii, reacting directly to free radicals [59]. Portes et al. [92] demonstrated that the exposure of the Toxoplasma parasite to dinuclear iron (III) compound reduced the activities of metalloenzymes, including SOD and CAT, which are important for the antioxidant protection of the parasite. This agent disturbs the redox balance of T. gondii, inducing parasite death in vitro, with no toxicity to the host cells. Moreover, Akerman et al. [57] demonstrated that Toxoplasma gondii is highly susceptible to oxidative stress that results from the disruption of its redox balance caused by tert-butyl hydroperoxide, juglone and phenazine methyl sulfate, with no effect on the host cells. Those compounds can affect the activity of peroxiredoxins, which are important in the protection of parasite cells against oxidative damage by hydroperoxides [57]. Another promising chemotherapeutic agent against T. gondii seems to be auranofin, which induces the accumulation of ROS in parasites via the inhibition of the thioredoxin reductase enzyme [93]. Thioredoxin reductase is essential for the survival of the parasite as maintaining a thioredoxin-dependent reduction state helps pathogens to resist oxidative-burst injury from host cells [55]. The results of Andrade et al. [94] revealed high efficiency of auranofin activity against T. gondii in vitro and in vivo, suggesting that it may be an effective alternative treatment for acute toxoplasmosis in the future. The induction of Toxoplasma parasite death due to increased ROS production was also reported as an impact of monensin treatment [56]. This drug is known to suppress the expression of the majority of T. gondii genes [95]. However, while monensin is used widely during anti-Toxoplasma therapy in animals, its toxicity precludes the use in humans.

The combination of pyrimethamine and sulfadiazine (the drugs of choice for toxoplasmosis) can suppress tachyzoite growth but has no effect on bradyzoites [96]. Therefore, most current therapies are ineffective against the latent stage of infection. Augusto et al. [65], using a CRISPR/Cas9 technique, successfully generated a genetic knockout of eIF2 kinase TgIF2K-B in the Toxoplasma parasite. As a result, the parasite failed to phosphorylate TgIF2α in response to oxidative stress, declining the ability to differentiate into tissue cysts. Those results may suggest that inhibition of TgIF2K-B may be a promising direction to develop new therapies based on diminished antioxidant protection, especially against persistent infection of T. gondii in its host.

### 4.2. Inorganic Nanoparticles

A possible source of alternative and effective agents in the treatment of Toxoplasma gondii infections may be also inorganic nanoparticles (NPs). The study of Adeyemi et al. [97] demonstrated that gold, silver, and platinum nanoparticles had promising in vitro anti-Toxoplasma activity without detectable host cell toxicity. The measurement through a fluorescent probe provided direct evidence that treatment with the use of nanoparticles promoted ROS production with a consequent contribution to parasite death. Other papers also show that silver nanoparticles (AgNPs), which have the broadest biomedical applications of all nanoparticles, may cause oxidative stress and DNA damage [98,99].

In studies on the impact of repeated administration of AgNPs on the antioxidant status of Wistar rats, an increase in lipid peroxidation and alteration of antioxidant enzymes were revealed in animal tissues and serum [98]. Thus, potential risks of host cell damage may be associated with the use of silver nanoparticles in the anti-Toxoplasma treatment. Hudecova et al. [99] confirmed oxidative DNA damage induced by AgNPs in human kidney cells in vitro. However, this oxidative damage was reported to be neutralized by pre-treatment with bioactive compounds, such as Gentiana extracts. An alternate solution may be the use of silver nanoparticles synthesized in combination with natural extracts of plants. Alajmi et al. [83] reported that AgNPs green synthesized with date palm and nabka exhibited some anti-Toxoplasma effects and significantly decreased the level of lipid peroxidation and NO concentrations with a concomitant increase in antioxidant enzymes activity in liver homogenate of mice infected with T. gondii.

Undoubtedly, the results of the presented studies support the idea that oxidative stress may be a good target for toxoplasmosis treatment. However, only limited data concerning oxidant–antioxidant effects of anti-toxoplasmosis treatment are available, and thus, further research is needed in this area.

## 5. Plant-Derived Antioxidants in Toxoplasmosis Treatment

The pathophysiology of toxoplasmosis is known to be related to the increased risk of oxidative stress in host cells due to the activation of inflammatory response against the parasite [85]. In chronic acquired toxoplasmosis, the level of MDA is elevated and can reflect indirectly the degree of oxidative cell damage [54]. Therefore, all molecules that diminish oxidative stress in the host cells may provide viable sources of alternative therapies for this parasitic infection (see Figure 2). The literature gives new insights into various natural extracts of plants with antioxidant properties, which may be good candidates for the discovery of novel drugs or may be useful as an alternative or adjuvant treatment option in the course of toxoplasmosis. It is worth mentioning that the current antimalarial drugs of choice (chloroquine and artemisinin derivatives) were derived from plants [100].

### 5.1. Tryptanthrin

An attractive candidate for the treatment of Toxoplasma gondii infections seems to be tryptanthrin. This natural alkaloidal compound and its derivatives are suggested to be inhibitors of T. gondii [101]. Considering parasite growth inhibition and host cell cytotoxicity, Krivogorsky et al. [102] demonstrated that, in particular, alcohol analogs are promising targets for further investigation as effective regimens. Tryptanthrin is considered a potential therapeutic agent mainly because of its structural simplicity and easy synthesis utilizing different starting materials and methods [103]. Moreover, tryptanthrin seems to be well absorbed across the intestinal epithelial cell layer, and its transepithelial transport is dominated by passive diffusion [104]. Moon et al. [105] demonstrated reduced oxidative damage of liver cells as a result of tryptanthrin pre-treatment, which effectively decreased the production of ROS and mitochondrial dysfunction. The antioxidant potential of tryptanthrin may be implemented via down-regulating the expression of nitric oxide synthase and thus the reduction of the amount of NO [103]. NO is well known to act as a neurotoxin by interacting with thiol groups of the proteins and decomposing into the highly reactive hydroxyl radicals [106]. The role of high NO production in the neuropathology of Toxoplasma encephalitis was revealed by Dincel et al. [107]. The authors suggested that NO overproduction led to neurotoxicity and acute degeneration, probably due to the stimulation of bradyzoite forms and reactivation of tissue cysts. 

### 5.2. Resveratrol and Selenium Compounds

The therapeutic effect in T. gondii-infected mice by reduction of the number of cysts in the brain and of pathological changes in the brain and liver was also revealed for a well-known antioxidant agent—resveratrol in combination with classic sulfamethoxazole-trimethoprim [108]. The authors demonstrated that a 10-day resveratrol therapy reduced the level of advanced oxidation protein products (AOPP) and the total oxidation status (TOS), which testifies the antioxidant properties of this therapy. Hence, the positive effect of co-administration of resveratrol with ST may be attributed to the inhibition of free radical production associated with toxoplasmosis pathology and the oxidative effect of typical chemical treatment. Moreover, Barbosa et al. [109] observed that co-administration of ST with selenium compounds, including diphenyl diselenide and sodium selenite, enhances the therapeutic effect in experimental toxoplasmosis in mice by reducing the lipid peroxidation and protein oxidation, providing a beneficial balance between the production of pro- and anti-inflammatory cytokines. The antioxidant effect of supplementation with diphenyl diselenide (PhSe)_2_ in T. gondii-infected mice, probably due to the activity of PhSe_2_ mimicking the enzymatic activity of GPx, was also confirmed by Machado et al. [70]. 

### 5.3. Herb Extracts

In addition to numerous studies on animal models, some natural compounds that exert both antioxidant and anti-Toxoplasma action were investigated in studies in vitro. In the study of Anacleto-Santos et al. [89], human cells were used to host T. gondii tachyzoites. The cells were exposed to the Pleopeltis crassinervata (fern) hexane fraction, which was found to contribute to the reduction of T. gondii tachyzoites viability, with no toxic effects to the host cells. Moreover, this is the first report of the antioxidant properties of P. crassinervata fronds. The moderate antioxidant and anti-Toxoplasma activity with no toxic effect to the studied cells was also observed for the essential oil isolated from guava leaves [110] and for Aloe vera and Eucalyptus methanolic extracts [111]. The antioxidant action of those natural compounds in the course of toxoplasmosis has a beneficial effect against pathological lesions caused by the presence of ROS. In addition, Eucalyptus showed antioxidant action in vivo, which resulted in a better survival rate of mice infected with Toxoplasma gondii [111]. The inhibition of T. gondii growth in vitro was also observed after exposure to Cola gigantea seed oil [112]. The authors of this research imply that C. gigantea presumably did not induce the generation of ROS, but the restriction of parasite growth is attributed to alternative ways. Moreover, those results confirmed the in vitro antioxidant potential of C. gigantea oil extract, which could be employed for biomedical applications. The beneficial effect on host cells infected with T. gondii was also reported for Eurycoma longifolia root extract [113] and aqueous extracts of neem and cinnamon leaves [114]. This effect was associated with antioxidant properties and concomitant anti-Toxoplasma activity of these extracts.

### 5.4. Ursolic Acid Derivatives

The potent anti-Toxoplasma activity both in vitro and in vivo was also revealed for ursolic acid derivatives, especially for the amide of the oxidized (C-3 oxo group) form of ursolic acid and 1H-tetrazol-5-amine, the compound named 12a [115]. Those compounds are possible inhibitors of TgCDPK1, which is an essential regulator of exocytosis in Toxoplasma and provides a mechanistic link between calcium signaling and motility but has no direct effect on the oxidant–antioxidant balance [116,117]. Nonetheless, the MDA level was significantly reduced in infected mice treated with ursolic acid derivatives (12a, 12b and 14a), indicating that these agents are able to decrease the lipid peroxidation process caused by acute T. gondii infection [115]. 

The oxidant–antioxidant effects of chemical compounds with anti-Toxoplasma activity are summarized in Table 1 (in vitro studies) and in Table 2 (animal models).

## 6. Melatonin and Lipid-Soluble Vitamins in Toxoplasmosis Treatment

Melatonin and vitamins D and E deserve special attention among the natural substances with known antioxidant properties when considering novel methods of treating toxoplasmosis. As lipid-soluble compounds, they can cross the brain-blood barrier, which seems to make them good candidates for the prevention and treatment of the cerebral form of toxoplasmosis. 

### 6.1. Melatonin Functions and Antioxidant Properties

Melatonin (N-acetyl-5-methoxytryptamine), a hormone produced mainly by the pineal gland, regulates circadian and seasonal rhythms and exerts numerous pleiotropic effects [118,119]. The synthesis and secretion of melatonin are under the control of the light/dark cycle, with the highest levels during the dark phase [118]. In view of reduced endogenous synthesis of melatonin with age and/or as a result of a disturbed circadian rhythm characteristic of the modern lifestyle, supplementation with this indoleamine is considered in numerous pathological conditions [120,121]. As an endocrine, paracrine and autocrine hormone, melatonin acts through membrane G protein-coupled receptors, nuclear receptors and calmodulin [122].

Moreover, melatonin, due to its lipid solubility, can cross cell membranes, interacting directly with intracellular proteins. It can also function in a receptor-independent manner through its free radical scavenging action [118]. It was confirmed that melatonin is able to neutralize ROS and RNS molecules, including H_2_O_2_, ^1^O_2_, O^2•−^, peroxynitrite (ONOO^−^) and OH^•^ [118]. As a water- and lipid-soluble molecule, melatonin acts both in the aqueous environment of the cells and body fluids and in plasma membranes. It was also found that melatonin may stimulate the expression of genes encoding for some antioxidant enzymes, including SOD, GPx and GR [118]. A wide range of melatonin physiological functions was described, including anti-inflammatory and immunomodulatory actions [119,120]. Melatonin was found to be a pleiotropic immunomodulatory agent acting through multiple mechanisms, such as promoting T cell differentiation, regulating cytokine gene expression, stimulating cytokine production in human peripheral blood mononuclear cells, activating an early phase of inflammation and suppressing chronic inflammation [121]. These properties make melatonin a molecule beneficial in viral, bacterial and parasitic infections [123]. 

### 6.2. Animal Model Studies on Melatonin in Toxoplasmosis

It was found that melatonin may stimulate the host immune response against the parasite in the course of various protozoan parasite infections [121,123,124]. In experimental models of Trypanosoma cruzi and Leishmania spp. infections, melatonin was proved to reduce parasitemia [124,125,126]. The beneficial effects of melatonin in malaria were also investigated [121,124,125]. However, few studies were conducted to assess the effect of melatonin in toxoplasmosis. 

In animal model studies, the effects of exogenous melatonin on the immune response of Toxoplasma-infected rats were assessed [127,128,129]. Melatonin was supplemented (3 mg/kg) in the rat model of Toxoplasma gondii retinochoroiditis [127]. In the experiment, several groups of infected animals were used, including pinealectomized rats and rats on a zinc-deficient diet with the zinc or/and melatonin supplementation. In the group of infected rats treated with both melatonin and zinc, the highest amount of cellular infiltration by lymphocytes, CD3+, CD4+, and CD8+ cells in the choroid and retina was observed. Single melatonin supplementation had no significant effect on the cellular infiltration in the choroid and retina in the rats after pinealectomy, whereas combined therapy improved the immune response. These results suggest that melatonin may increase the absorption of zinc, which seems to be a mediator of the melatonin impact on the immune system [124]. Similarly, Baltaci et al. [128] found that melatonin and zinc supplementation activated cellular immunity by stimulating the total lymphocyte, CD4+ and CD8+ production in Sprague-Dawley male rats infected with T. gondii. Moreover, in the pinealectomized rats infested with T. gondii, the cellular immunity was significantly reduced when compared to the control rats with toxoplasmosis [129]. 

It is worth mentioning that Toxoplasma-infected rats had increased plasma nitrite levels, and in pinealectomized rats with toxoplasmosis, this increase was more pronounced [130]. This melatonin deficiency effect may be related to its antioxidant properties as described above, including the direct ROS scavenging ability and the induction of the synthesis of antioxidant enzymes. Moreover, melatonin can reduce the activity of induced NO synthase, reducing in this way the production of NO. As was mentioned above, elevated blood plasma levels of NO in the course of toxoplasmosis have an immunoprotective and immunomodulatory effect, but the overproduction of NO as a result of its neurotoxic action leads to CNS damage [131]. Taking into account these findings, melatonin appears to be a very promising CNS protection agent in patients with toxoplasmosis.

### 6.3. In Vitro Studies on Melatonin in Toxoplasmosis

To our best knowledge, there exist only two studies concerning the direct effects of melatonin on Toxoplasma development after in vitro infection of host cells. In the research of Machado et al. [132], the effects of melatonin treatment in the monkey kidney cell epithelial line LLC-MK2 after infection with T. gondii were analyzed. Melatonin reduced parasite proliferation in the LLC-MK2 cells at 24 and 48 h and at 6 days. The IC_50_ of melatonin to inhibit T. gondii growth was 3 mM at 24 h, 1.69 mM at 48 h and 1.13 mM at 6 days. Under the transmission electron microscope, tachyzoites with an altered shape, ruptured plasma membranes and cytoplasmic leakage, characteristic of necrotic cell death, were seen. The host cells presented numerous vacuoles, probably due to the digestion of parasites. Moreover, the parasites showed positive staining for apoptotic-like cell death. The authors concluded that melatonin might induce parasite cell death through both necrosis and apoptosis, pointing to changes in the energy metabolism of the parasite. The results of the study strongly indicate that melatonin can permanently inhibit T. gondii growth at 48 h posttreatment. It was observed that even after 6 days of melatonin treatment, there was no formation of tissue cysts, which suggests that melatonin may prevent conversion into bradyzoites and the formation of tissue cysts. 

In the study of Majumdar et al. [133], 0.2 mM melatonin was used to assess its antioxidant effects in the Toxoplasma-infected human intestinal epithelial cell line Caco2. They found that T. gondii reduced the H_2_O_2_ levels produced in the host cells with the use of tert-butyl hydroperoxide. This reduction was greater in melatonin-treated cells. As a result, the parasite growth was augmented. The concentration of melatonin used in the experiment did not induce significant cytotoxicity, and more than 90% of the cells were viable. Moreover, the authors suggested that T. gondii is able to catabolize tryptophan into melatonin, thus prolonging the survival of infected cells. Therefore, tryptophan catabolites and their analogues might be used as effective therapeutic molecules to impair the regulatory processes leading to melatonin synthesis in the parasite. It should be underlined that the melatonin concentration used in the Majumdar et al. [133] study was about ten times lower than in the Machado et al. [132] experiment. Undoubtedly, melatonin metabolism in Toxoplasma gondii and in host cells requires further investigations. The results of the existing studies strongly support the use of melatonin in an alternative, safe and effective treatment for toxoplasmosis. However, further research is needed to analyze this possibility and to understand the mechanisms of melatonin action in toxoplasmosis.

### 6.4. Vitamin D Structure and Functions

Vitamin D is a term related to a group of secosteroids, among which 1,25-dihydroxycholecalciferol (1,25(OH)_2_D_3_; calcitriol) has the highest biological activity [134]. Two forms of vitamin D, namely cholecalciferol (vitamin D3) and ergocalciferol (vitamin D2), can be found in food [135]. However, the most significant source of cholecalciferol in the human organism is its synthesis in the skin from 7-dehydrocholesterol as a result of ultraviolet irradiation [135]. Due to the subsequent action of enzymes present in the liver and kidney, the active hormone calcitriol is produced [134]. Vitamin D deficiency is a growing problem worldwide, reaching about 30%–50% on a global scale, especially in elderly people [136]. Calcitriol is a lipid-soluble hormone that acts through its nuclear receptor (vitamin D receptor, VDR) that functions as a transcription factor [134]. In addition to the regulation of calcium-phosphate metabolism, vitamin D was found to exert numerous pleiotropic actions, including the regulation of multiple cellular processes with effects on cell growth and differentiation, the innate and adaptive immune function, cardiovascular function, and others [134]. Vitamin D is considered to have antioxidant properties, but specific mechanisms have not yet been fully recognized [137]. It was proven that supplementation with vitamin D could improve parameters of oxidative stress, including reducing lipid peroxidation markers and increasing the level of GSH [137]. 

### 6.5. Studies on Vitamin D in Toxoplasmosis

Taking into account immunosuppressive, anti-proliferative and pro-apoptotic properties of calcitriol, the effect of 1,25(OH)_2_D_3_ on T. gondii-infected mice was investigated [138]. Surprisingly, it was reported that the treated mice were at higher risk of acute toxoplasmosis. On the other hand, lower parasitic burdens were found in the tissues, and mild pathological lesions were observed in the organs. To understand better the pathophysiological mechanisms of the observed processes, the same group of scientists performed a quantitative analysis in cell-based assays [139]. No difference in the number of infected cells incubated either in the presence or absence of 1,25(OH)_2_D_3_ was found. However, a significant dose-dependent inhibition with a potent inhibition of intracellular parasite growth at 10^−7^ M of calcitriol was described. According to these results, calcitriol does not appear to play any role in the invasion and/or aggression, but the inhibition of parasite growth may be a result of cellular effects mediated by 1,25(OH)_2_D_3_. Endogenous calcitriol was also found to decrease the humoral IgE and IgG1 response to parasite infection [140]. Similarly, in the case of Leishmania amazonensis, vitamin D was shown to increase susceptibility to parasite infection in mice, but in an in vitro study, the reduction of parasite growth in infected macrophages in a process independent of macrophage oxidative mechanisms was demonstrated [141]. In the case of Plasmodium spp., vitamin D was found to inhibit parasite growth extensively during the acute phase and subsequent parasite growth, forming a mildly delayed peak [142,143].

Interestingly, Ghaffarifar et al. [144] demonstrated that one dose of vitamin D3 given to mice before the infection with Toxoplasma tachyzoites could affect T. gondii proliferation and NO production by infected macrophages, whereas daily injections throughout one week might suppress the immune system. The positive effect of vitamin D3 was improved by using interferon-gamma (IFN-γ). In the study, it was also shown that the elevated production of NO might be related to the observed anti-parasitic effect of a single vitamin D3 injection. 

Data concerning vitamin D and toxoplasmosis in humans are limited. In a Women’s Reproductive Health Cohort Study performed from 2007 to 2010 in Henan Province of China, a higher risk of vitamin D deficiency in women of childbearing age infected with T. gondii was found, in comparison with uninfected women [145]. A similar result was obtained by Fakhrieh Kashan et al. [146], who a found higher prevalence of T. gondii infection in vitamin D deficient patients.

To summarize, in the light of existing studies, the use of vitamin D in the treatment of Toxoplasma seems to be problematic. The results of in vitro and in vivo studies are conflicting. It should be emphasized that high doses required to obtain positive immune effects in the host organism can result in severe side effects, including hypercalcemia [144]. Vitamin D deficiency was correlated with higher Toxoplasma infection prevalence, but the effects promoted by vitamin D in the immune system, including decreasing production of interleukin 12 (IL-12) and tumor necrosis factor-alpha (TNF-α) by monocytes and macrophages, and IFN-γ by CD4+ T cells, decreasing the differentiation of Th1 cells and increasing the differentiation of regulatory T cells, are unfavorable to the development of an effective immune response against the protozoan parasites [141]. Nevertheless, the in vitro studies revealed the potential of vitamin D in toxoplasmosis treatment. Unquestionably, further research is needed to understand the complexity of the vitamin D role in Toxoplasma gondii infection and treatment.

### 6.6. Vitamin E Functions and Antioxidant Properties

Another lipid-soluble substance considered in toxoplasmosis treatment is vitamin E. Eight compounds, including α-, β-, γ- and δ- tocopherols and α-, β-, γ- and δ- tocotrienols, found in food, are called vitamin E [147]. Among them, only α-tocopherol meets the vitamin E requirement in humans [148]. Vegetable oils and nuts are good sources of this vitamin. Vitamin E is considered to be a major lipid-soluble antioxidant, scavenging peroxyl radicals and terminating the oxidation of polyunsaturated fatty acids [147,148]. This function is critical to maintaining the bioactivity of plasma membranes and regulating various signaling pathways dependent on oxidative stress [148]. Vitamin E was proved to stimulate immune response in animal and human models against several infectious diseases [147]. Increased lymphocyte proliferation and natural killer (NK) cell activity, increased interleukin 2 (IL-2) production and immunoglobulin levels, as well as decreased interleukin 6 (IL-6) production were observed as a result of dietary interventions of vitamin E in animals and humans [147].

### 6.7. Studies on Vitamin E in Toxoplasmosis

There are a few publications on vitamin E and toxoplasmosis. In the Swiss study of 10,000 cord blood samples, 2.8% of specimens exhibited anti-Toxoplasma antibodies, suggesting acute or chronic infection [149]. In this study, significantly lower average tocopherol serum concentrations in 282 T. gondii-positive cases were found compared to 280 control samples. Correspondingly, in the study of blood donors in Egypt, the levels of alpha, gamma and lambda tocopherol fractions were lower in T. gondii-seropositive than in seronegative blood donors [82]. It was associated with higher MDA levels and decreased GPx activities. It was also observed that in T. gondii-seropositive school-age children, Toxoplasma-associated memory impairment was worse in children with lower serum vitamin E concentrations [150]. 

However, in the study of McCarthy and Davis [151], it was found that Swiss Webster or C57B1/6J mice infected with oocysts of T. gondii and supplemented with vitamin E and/or selenium, an element synergistic to vitamin E, had an increased number of tissue cysts, tissue pathology and weight loss. A diet deficient in vitamin E and/or selenium led to a lower average number of tissue cysts and slight evidence of tissue pathology during chronic infection. It was consistent with some previous reports describing the protective effect of a pro-oxidant diet and vitamin E deficiency in animal models of malaria and Babesia sp. infection [151]. In the human study of Olofin et al. [152], HIV-infected pregnant Tanzanian women were supplemented with multivitamins, including B complex, C, and E. The supplementation protected against the development of symptomatic malaria, but the increased risk of malaria parasitemia was observed among supplemented women. Interestingly, it was described that α-tocopherol might be used as an adjuvant molecule in a DNA vaccine for Toxoplasma gondii infections, contributing to the increased vaccination activity [153].

It could be concluded that the effect of vitamin E in toxoplasmosis is an issue requiring further carefully planned studies, as it was suggested in the case of vitamin D and melatonin. Only a few experiments have been performed, and the results are inconclusive and raise more questions than answers. Scientists should be encouraged to make further efforts to investigate possible mechanisms of vitamin E action in the course of toxoplasmosis and its complications.

## 7. Conclusions

In the course of Toxoplasma gondii infection, similarly to other intracellular protozoan parasites, oxidative stress in both the parasite and the host was proven to be one of the mechanisms involved. Nevertheless, the scrupulous analysis of the available literature data shows that the involvement of pro- and antioxidant processes in the development of the disease and in the treatment has not been thoroughly investigated. Surprisingly, among numerous studies concerning new therapies of toxoplasmosis, only a few studies examined the oxidant–antioxidant processes related to the course of the disease. Considering the importance of oxidative stress in the course of toxoplasmosis and the immune defense of the host, the search for drugs influencing the oxidant–antioxidant balance of the parasite seems to be a priority. Admittedly, there are many studies on improving the antioxidant properties of the host organism, especially by natural compounds, to defend the host against the adverse effects of oxidative stress associated with the Toxoplasma gondii infection. Interestingly, supplementation with some antioxidants was found to promote the increase in parasitemia, but the disease was then characterized by a milder course. Undoubtedly, more studies should be conducted in order to analyze the mechanisms of action. In particular, there is a need for the validation of in vitro and animal model results in human studies.

## Figures and Tables

**Table 1:** The effect of selected therapeutic agents on *T. gondii* viability and the oxidant–antioxidant balance in the parasite and in the host in in vitro studies.

Models	Compound	IC_50_ /EC_50_	Results/Oxidant–Antioxidant Effect	References
Human foreskin fibroblasts	*tert*-butyl hydroperoxide	IC_50_ = 100 ± 8 nM	• ↑ *T. gondii* parasites death • stable viability of the host cells • ↓ peroxiredoxin activity • changes in redox status of the parasites and destruction of their oxidant homeostasis	
	5-hydroxy-1,4-naphtoquinone (Juglon)	IC_50_ = 148 ± 6 nM		
	Phenazine methyl sulfate	IC_50_ = 406 ± 134 nM		[57]
Vero cell line from kidney of African green monkey	Extract of *Azadirachta indica* leaves	*Toxoplasma* elimination at concentrations higher than 2 mg/mL	• ↑ disorganization and elimination of intracellular tachyzoites • antioxidant effect on host cells	[114]
	Extract of *Melia azedarach* leaves	*Toxoplasma* elimination at concentrations higher than 0.5 mg/mL	• ↑ disorganization and elimination of intracellular tachyzoites • antioxidant effect on host cells	[113]
Human foreskin fibroblasts	6-oximes of the tryptanthrin (6,12-dihydro-6,12-dioxoindolo-(2,1-*b*)-quinazoline) and its 8-bromoderivative	ID_50_ range (0.003 ÷ 1.3) µM	• anti-*T. gondii* activity • no host cell cytotoxicity • hepatoprotection of host cells • activation of the ERK-Nrf2 pathway • ↓ oxidative damage	[101,102,105]
	6-hydrazones of the tryptanthrin and its 8-bromoderivative	ID_50_ range (0.31 ÷ 12) µM		
	6-hydroxy derivatives of the tryptanthrin and 8-bromotryptanthrin	ID_50_ range (0.002 ÷ 8.7) µM		
Vero cell line from kidney of African green monkey entry 4	Extract of *Psidium guajava* (guava)	EC_50_ = 4.94 ± 0.039 µg/mL	• inhibition of *T. gondii* growth • no host toxicity • moderate antioxidant—inhibition of free radicals action in host cells	[110]
Human foreskin fibroblasts	1-thio-*β*-D-glucopyranosatotriethylphosphine gold-2,3,4,6-tetraacetate (Auranofin)	IC_50_ = 0.28 nM	• ↓ viability of *T. gondii* parasites • inhibition of thioredoxin reductase activity • ↓ resistance to oxidative damage	[55,93,94]
LLC-MK2 host cells—kidney epithelial cells of rhesus monkey	[Fe(1-(*bis*-pyridin-2-ylmethyl-amino)-3-chloropropan-2-ol)(SO_4_)]_2_ *µ*-oxo	IC_50_ = 3.6 µM nontoxic up to 200 µM	• inhibition of parasites growth at concentration for 2.5–25 µM • no effect on host cell viability • ↑ oxidative stress • ↓ metalloenzymes activity	[92]
Human foreskin fibroblasts	Gold nanoparticles (AuNPs)	EC_50_ ≤ 7 µg/mL	• ↓ viability of the parasites • no host toxicity • alteration in redox status by ROS production and reduction in mitochondrial membrane potential	[97]
	Silver nanoparticles (AgNPs)	EC_50_ ≤ 1 µg/mL		
	Platinum nanoparticles (PtNPs)	EC_50_ ≤ 100 µg/mL		
Vero cell line from kidney of African green monkey	Extract of *Aloe vera*	IC_50_ = 13.2 µg/mL	• anti-*T. gondii* activity in infected cells • ↓ MDA levels in host cells • potent antioxidant effect	[111]
	Extract of *Eucalyptus globulus*	IC_50_ = 24.7 µg/mL		
Human foreskin fibroblasts	Seed oil of the fruit of *Cola gigantea*	EC_50_ ≤ 15 µg/mL	• ↓ viability of *T. gondii* parasites • ↑ toxicity for parasites by ROS production • mild antioxidant potential in host cells	[112]
GES-1 cells	Amide of 10-oxo derivative of ursolic acid and 1-*H*-tetrazol-5-amine	IC_50_ = 218.6 µM	• reduction of number of *T. gondii* invading of host cells • antioxidant effect on host cells	[115]

EC_50_, half maximal effective concentration.; ERK-Nrf2, extracellular signal-regulated kinase-Nuclear factor-E2 p45-related factor; GES-1 cells, human gastric epithelial cell line; IC_50_, half maximal inhibitory concentration; ID_50_, infective dose; MDA, malondialdehyde; ROS, reactive oxygen species. In column 4, the anti-*T. gondii* activities of the therapeutic agents are marked in blue, and their specific oxidant-antioxidant effects in black.

**Table 2:** The effect of selected therapeutic agents on *T. gondii* viability and the oxidant–antioxidant balance in the parasite and in the host in animal models.

Models	Compound	Dosage	Results/Oxidant–Antioxidant effect	References
Balb/c mice	Sulfamethoxazole/trimethoprim(S/T) supplemented with diphenyl diselenide or sodium selenite	S/T—0.5 mg/kg bid orally Na_2_O_3_Se—1 mg/kg intramuscularly	• ↓ viability of the parasites • ↓ TBARS and AOPP in liver samples • ↓ lipid peroxidation and protein oxidation in host organism	[109]
		S/T—0.5 mg/kg bid orally Ph_2_Se_2_—5 µmol/kg subcutaneously		
Fourteen day old chicken embryos	1-thio-*β*-D-glucopyranosatotriethylphosphine gold-2,3,4,6-tetraacetate (Auranofin)	1 mg/kg a single dose	• protection from death in all acutely infected embryos • reduction of the parasite load in organs such as brain and liver • inhibition of thioredoxin reductase activity • ↓ resistance to oxidative damage	[55,93,94]
Female mice	Diphenyl diselenide	5 µmol/kg subcutaneously	• no reverse of the behavioral changes caused by the parasite • ↓ TBARS and ↑ GST in brain of the host • protective action as an antioxidant	[70]
Female Balb/c mice	Extract of *Aloe vera*	50/100 mg/kg/day orally	• ↑ survival rate of infected mice • ↓ MDA levels in host cells • potent antioxidant activity	[111]
	Extract of *Eucalyptus globulus*	100/200 mg/kg/day orally		
Female Balb/c mice	Silver nanoparticles (AgNPs) green synthesized by *Phoenix dactylifera* (date palm) extract and *Ziziphus spina-christi* (Nabka) powder	100 mg/kg/day orally	• ↑ survival rate of infected mice • ↓ lipid peroxidation, ↓ NO concentration in liver homogenate • ↑ GSH, SOD, CAT in liver homogenate	[83]
Female KM mice	Amide of 10-oxo derivative of ursolic acid and 1-*H*-tetrazol-5-amine	100 mg/kg/day oral gavage	• inhibition of tachyzoite growth in infected mice • restoration of the normal body weight of infected mice and reduction of hepatotoxicity • ↓ MDA levels and ↑ GSH • ↓ lipid peroxidation	[115]

AOPP, advanced oxidation protein products; CAT, catalase; GSH, reduced glutathione; GST, glutathione S-transferase; MDA, malondialdehyde; NO, nitric oxide; SOD, superoxide dismutase; TBARS, thiobarbituric acid reactive substances. In column 4, the anti-*T. gondii* activities of the therapeutic agents are marked in blue, and their specific oxidant-antioxidant effects in black.

## Data Availability

Data sharing not applicable. No new data were created or analyzed in this study. Data sharing is not applicable to this article.
